# Computational Pipeline for NIRS-EEG Joint Imaging of tDCS-Evoked Cerebral Responses—An Application in Ischemic Stroke

**DOI:** 10.3389/fnins.2016.00261

**Published:** 2016-06-20

**Authors:** Debarpan Guhathakurta, Anirban Dutta

**Affiliations:** ^1^School of Clinical Sciences, University of BristolBristol, UK; ^2^Department of Psychology and Neurosciences, IfADo – Leibniz Research Centre for Working Environment and Human FactorsDortmund, Germany

**Keywords:** transcranial direct current stimulation, near-infrared spectroscopy, electroencephalography, neurovascular coupling, computational modeling, ischemic stroke

## Abstract

Transcranial direct current stimulation (tDCS) modulates cortical neural activity and hemodynamics. Electrophysiological methods (electroencephalography-EEG) measure neural activity while optical methods (near-infrared spectroscopy-NIRS) measure hemodynamics coupled through neurovascular coupling (NVC). Assessment of NVC requires development of NIRS-EEG joint-imaging sensor montages that are sensitive to the tDCS affected brain areas. In this methods paper, we present a software pipeline incorporating freely available software tools that can be used to target vascular territories with tDCS and develop a NIRS-EEG probe for joint imaging of tDCS-evoked responses. We apply this software pipeline to target primarily the outer convexity of the brain territory (superficial divisions) of the middle cerebral artery (MCA). We then present a computational method based on Empirical Mode Decomposition of NIRS and EEG time series into a set of intrinsic mode functions (IMFs), and then perform a cross-correlation analysis on those IMFs from NIRS and EEG signals to model NVC at the lesional and contralesional hemispheres of an ischemic stroke patient. For the contralesional hemisphere, a strong positive correlation between IMFs of regional cerebral hemoglobin oxygen saturation and the log-transformed mean-power time-series of IMFs for EEG with a lag of about −15 s was found after a cumulative 550 s stimulation of anodal tDCS. It is postulated that system identification, for example using a continuous-time autoregressive model, of this coupling relation under tDCS perturbation may provide spatiotemporal discriminatory features for the identification of ischemia. Furthermore, portable NIRS-EEG joint imaging can be incorporated into brain computer interfaces to monitor tDCS-facilitated neurointervention as well as cortical reorganization.

## Introduction

Transcranial direct current stimulation (tDCS) is a non-invasive brain stimulation technique that involves application of low intensity direct currents at the scalp for the modulation of central nervous system excitability in humans (Woods et al., 2016). Beyond effects on neuronal excitability after-effects of tDCS on regional cerebral blood flow (rCBF), i.e., increase in resting state rCBF after anodal tDCS and decrease after cathodal tDCS, have been demonstrated (Zheng et al., 2011). Changes in rCBF can be related to the energetic demand of local neuronal activation, which is termed neurovascular coupling (NVC; Girouard and Iadecola, 2006). Although the proposition of a correlation between neuronal activity and the increment of vascular supply due to the brain's energy demand increase is a long-standing concept (Roy and Sherrington, 1890): “…the brain possesses an intrinsic mechanism by which its vascular supply can be varied locally in correspondence with local variations of functional activity,” the exact cellular mechanism of NVC is still elusive (Girouard and Iadecola, 2006). The importance of NVC to the health of the normal brain has been highlighted in a review by Girouard and Iadecola (2006) that suggested it as a therapeutic target in pathologies associated with cerebrovascular dysfunction. Pulgar (2015) has proposed tDCS for improvement of cerebrovascular dysfunction, based on findings showing that it modulates cerebral vasomotor reactivity (VMR), and heart rate variability (Vernieri et al., 2010). This indicates widespread effects of tDCS on human NVC, VMR, and cerebral autoregulation (List et al., 2015). However, the effects depend on the tDCS electrode montage, e.g., List et al. (2015) showed with a double-blind crossover within-subject design that 20 min of anodal tDCS with cephalic stimulation and return electrodes did not affect the cerebral autoregulation assessed by low-frequency oscillations (LFO) of cerebral blood flow. Therefore, they hypothesized that the extracephalic return electrode in the study by Vernieri et al. (2010) may have stimulated the brainstem autonomic centers which can be easily assessed with the calculations of electric field (and current density) induced by tDCS (Noetscher et al., 2014). Moreover, the mechanistic understanding of the interactions between neuronal and hemodynamic responses to tDCS due to NVC is largely unknown in health and disease (Dutta, 2015).

In this methods paper, we present a portable multi-modal neuroimaging method involving near-infrared spectroscopy (NIRS; Muehlschlegel et al., 2009; Obrig, 2014) and electroencephalography (EEG; Buzsáki et al., 2012; Foreman and Claassen, 2012) to monitor interactions between neuronal and hemodynamic responses to tDCS. NIRS-EEG joint imaging might be relevant in ischemic stroke or cerebrovascular accident, which affects 15 million people worldwide (WHO | The Atlas of Heart Disease and Stroke)^1^. Ischemic stroke is caused due to obstruction of blood supply to a brain area resulting in a corresponding loss of neurologic function, constituting about 87% of all stroke cases (Go et al., 2013). NIRS-EEG joint imaging of tDCS responses may be used to identify and assess ischemic brain areas (Dutta et al., 2015; Jindal et al., 2015b) for neurointervention, and such online portable neuroimaging technique incorporated into brain computer interfaces may enable customized dosing of tDCS for the improvement of cerebrovascular function (Dutta, 2015; Pulgar, 2015) in cerebrovascular occlusive disease.

### Hypothesized role of neurovascular coupling function during transcranial direct current stimulation (tDCS)

Neurovascular coupling (NVC) is defined by neural activity closely related, spatially and temporally, to rCBF. The neurovascular unit (NVU) consists of the endothelium, glia, neurons, pericytes, and the basal lamina (Dutta, 2015). NVC dysfunction may disrupt rCBF and metabolic regulation. Computational models can capture NVU dynamics (Huneau et al., 2015). However, no general pattern of dysfunction of glial-neuronal interactions has been developed in experimental brain disorders (Kondziella, 2005). Nevertheless, the interpretation of disorder-related changes in cortical neural and hemodynamic activity in terms of the energetics of excitation and inhibition critically relies on the functional and metabolic interactions between neurons and astrocytes, e.g., the glutamate/glutamine and GABA/glutamine neurotransmitter cycle (Patel et al., 2005), that may be disturbed (Kondziella, 2005). Therefore, disorder-specific computational models are required. Simple low-dimensional models can describe NVU as a lumped system to relate neural activity with an “energy” variable (analogous to ATP) as output (Chhabria and Chakravarthy, 2016). ATP is required for neuronal metabolic processes like synapto-vescicular recycling and maintenance of the gradient potential (Hamel, 2006; Attwell et al., 2010).

The constant supply of ATP using oxygen and glucose is mediated by the regulation of large cerebral arteries and cerebral microvascular pressure (Faraci and Heistad, 1990) via brain's vascular properties, e.g., arteriolar smooth muscle cells contractility (Hill et al., 2015). Here, bidirectional interactions between neuronal and hemodynamic cerebral responses to tDCS were postulated (Dutta, 2015). Specifically, tDCS-evoked increases of neuronal activity might result in aerobic glycolysis (Vaishnavi et al., 2010) and associated lactate surge (Mintun et al., 2004) which can modulate spatiotemporal activity of primary cortical neurons through a receptor-mediated pathway (Bozzo et al., 2013). Besides the role of lactate in energy metabolism, a signaling molecule inducing calcium influx and the expression of long-term plasticity-related genes in neurons has recently been identified (Yang et al., 2014). Initially, neuronal membranes are polarized by tDCS that may alter the spontaneous activity with no effects on synaptic plasticity (Stagg and Nitsche, 2011). Such altered neuronal states may affect rCBF by hyperpolarizing or depolarizing vascular smooth muscles causing vasoconstriction or vasodilation via various metabolites like K+, adenosine, NO, or CO2 (Dutta, 2015). Four kinds of potassium channels, namely ATP-sensitive potassium channels, calcium-activated potassium channels, delayed rectifier potassium channels, and inward rectifier potassium channels play the major role in maintenance of vascular tone of cerebral blood vessels. Via activation of these channels, efflux of K+ causes closure of voltage-dependent calcium channels leading to vascular relaxation (Nelson et al., 1990; Bonnet et al., 1991; Edwards and Weston, 1993; Kitazono et al., 1995; Brayden, 1996). Also, neuronal Nitric Oxide Synthase (NOS) plays a significant role in maintenance of cerebral blood flow (Attwell et al., 2010). Activation of neuronal NMDA receptors via glutamate causes an influx of calcium that activates NOS and increases blood flow (Attwell et al., 2010). Indeed, the aftereffects of anodal tDCS depend on the modulation of both GABAergic and glutamatergic synapses and are calcium-dependent (Stagg and Nitsche, 2011). Moreover, astrocytes, that are proposed to be powerful regulators of neuronal spiking, synaptic plasticity and brain blood flow (Bazargani and Attwell, 2016), are involved in the generation of calcium waves between neighbored neurons via metabotropic glutamate receptors (Leybaert et al., 1998). Astrocytes regulate increased local blood-flow during neuronal activation (high energy demand) by secretion of vasoactive substances like NO, and Prostaglandin E2 that are involved in synaptic plasticity (Oomagari et al., 1991; Leybaert et al., 1998). Anatomical connections between the vascular system and astrocytes at the functional level are well-known (Mathiisen et al., 2010). Astrocytes express a surface protein required to detect neuronal activation and facilitate the gated efflux of K+ that causes vasodilation (Paulson and Newman, 1987). Our recent understanding of astrocyte calcium signaling and its relation to neuronal function (Bazargani and Attwell, 2016) can metabolically bind NVU to NVC mechanism as well as its dysfunction (Bélanger et al., 2011), e.g., how vasomotion rhythms influence neural firing patterns (Nikulin et al., 2014) in health and disease.

In principal accordance, tDCS-evoked cerebral responses in disease states may provide spatiotemporal discriminatory features of dysfunction, e.g., in acute ischemic stroke, transient ischemic attack, vascular dementia, etc. Alterations of neuronal state and excitability are reflected in EEG (Dutta and Nitsche, 2013; Ferreri et al., 2014; Bailey et al., 2016) while alterations of the hemodynamic state and mitochondrial dysfunction can be captured with NIRS (Cooper et al., 1999; Obrig, 2014). Simultaneous recordings of EEG, NIRS, arterial blood pressure, respiration, etc. (Nikulin et al., 2014) may be relevant for identifying discriminatory features or biomarkers of dysfunction in cerebrovascular diseases. In this method paper, we focus on NIRS-EEG joint imaging of NVC function using tDCS-evoked cerebral responses in ischemia.

### NIRS-EEG joint imaging of tDCS-evoked cerebral response in ischemia

During ischemia, as rCBF declines, protein synthesis is inhibited at a threshold of about 0.55 ml/gm/min, anaerobic glycolysis starts at around 0.35 ml/gm/min, energy metabolism is disturbed from around 0.20 ml/gm/min, and then anoxic depolarization starts when the rCBF falls below 0.15 ml/gm/min (Hossmann, 1994). CBF and EEG alterations are highly correlated (Sharbrough et al., 1973; Foreman and Claassen, 2012) with major change below 0.17 ml/gm/min, moderate change between 0.18 and 0.30 ml/gm/min, and no change above 0.30 ml/gm/min. In the zone of ischemic penumbra, i.e., the cerebral zone for which perfusion is < 0.20 ml/gm/min, deprivation of oxygen-glucose to the neurons due to interrupted blood supply leads to unavailability of vital energy required to maintain neuronal transmembrane ion gradients (Pellerin and Magistretti, 2004). This in turn increases overall neuronal depolarization (increased extracellular K+ levels) and release of excitatory neurotransmitters, including a recycling pathway which increases intracellular levels of Na+ and Ca2+, and activates cellular signaling pathways responsible for neuronal damage and cell death (Szydlowska and Tymianski, 2010; Sabogal et al., 2014). In such a pathological brain condition, functional and structural damage to cerebral homeostasis maintaining NVU causes loss of functional interaction between neural activity and vascular architecture contributing to deterioration of cerebral autoregulation and neurovascular coupling following stroke onset (Salinet et al., 2014). Severe perfusion deficits cause sustained depolarization that increases extracellular Glutamate concentration leading to neuronal cell death primarily due to mitochondrial dysfunction (damaged ATP synthesis, increased depolarization, hyper-stimulation of proteases; Greenwood and Connolly, 2007). This functional and structural damage also results in detrimental physiological conditions of increased permeability of the blood-brain-barrier (a part of NVU), loss of cerebral auto-regulation, oxidative stress and inflammation (Schoknecht et al., 2015).

In principal accordance, NIRS-EEG joint imaging under tDCS perturbation may be useful to detect NVU state in ischemia with specificity (Jindal et al., 2015b), and subsequent excitability reducing cathodal tDCS (Notturno et al., 2014) and/or mitochondrial function (i.e., increased ATP production) promoting photobiomodulation (Hashmi et al., 2010) may be neuroprotective before the injury becomes irreversible. Moreover, it may be possible to measure cytochrome oxidase redox state changes in the brain noninvasively indicating mitochondrial dysfunction (Cooper et al., 1999) besides oxygenated and deoxygenated hemoglobin concentration using multi-wavelength NIRS (Dunne et al., 2014). Here, parameter estimation using online NIRS-EEG joint imaging data with computational NVU models under normal and energy-starved conditions (Chhabria and Chakravarthy, 2016) can be used to identify, monitor, and therapeutically target ischemic brain areas (Jindal et al., 2015a).

## Methods

In this methods paper, we present a software pipeline for NIRS-EEG joint imaging of tDCS-evoked cerebral responses. The software pipeline incorporates freely available SimNIBS (Windhoff et al., 2013) for calculations of electric fields (and current density) induced by tDCS which was used by prior work (Datta et al., 2011) to determine tDCS-affected brain areas. The software pipeline incorporates the headModel module of the open-source MoBILAB toolbox (Ojeda et al., 2014) for computing EEG forward and inverse solutions to identify EEG scalp topography that can record from tDCS-affected brain regions. The software pipeline incorporates also the probe design module of the freely available AtlasViewer software (Aasted et al., 2015) to compute NIRS forward models for developing source and detector probe geometry to cover tDCS-affected brain regions. For demonstration, we applied this software pipeline on the Colin27 average brain—a stereotaxic average of 27 T1-weighted MRI scans of the same individual (Holmes et al., 1998). Based on our prior work (Dutta et al., 2015), we also present in the last section a cross-correlation analysis approach to capture the coupling relation between regional cerebral hemoglobin oxygen saturation and the log-transformed mean-power time-series from EEG in the case of a chronic (>6 months) ischemic stroke survivor (76 years old male with left MCA stroke in 2011).

### An MRI based head model

We leveraged the freely available SimNIBS software pipeline (Windhoff et al., 2013) to develop subject-specific head model based on MRI data. SimNIBS incorporates FreeSurfer tools (Fischl, 2012) to segment the brain and FSL (Jenkinson et al., 2012) BET/BETsurf tools to segment the rest of the head. Developers of the SimNIBS software pipeline recommend MPRAGE acquisitions with selective water excitation for fat suppression for FreeSurfer tools to work well (version2:mri_sequences | SimNIBS)^2^. For FSL BET/BETsurf tools, they recommend high bandwidths both for the T1- and T2-weighted images and thin slices with gaps in-between for the T2-weighted images. Therefore, ideally four sets of images should be acquired, two with fat suppression and two without fat suppression, but with high bandwidth and thin slices. The SimNIBS software pipeline (Windhoff et al., 2013) will then use the fat-suppressed T1 as input for FreeSurfer, the fat-suppressed T1- and T2-weighted images to reconstruct the inner skull boundary, and the normal T1- and T2-weighted images to reconstruct the outer skull boundary and the skin surface with FSL (Jenkinson et al., 2012) BET/BETsurft tools. This software pipeline was applied on the Colin27 average brain, which is based on 27 times on an individual, and linear registration of the images to create an average with high SNR and structure definition (Holmes et al., 1998). The tetrahedral head meshes from the Colin27 average brain MRI data were generated using the “mri2mesh” tool in the SimNIBS software pipeline (Windhoff et al., 2013) for our MRI-based head model.

### A computational approach to target vascular territories with tDCS

Computational approaches are available to optimize multi-channel tDCS to target specific brain regions (Otal et al., 2016). The goal of this project is to selectively target cerebral vascular territories that may be affected by ischemic stroke. We present this approach for bipolar tDCS to target primarily the outer convex brain territory of the MCA (Dutta et al., 2015). A PISTIM electrode (Neuroelectrics NE, Barcelona, Spain) with π cm^2^ (1 cm radius) contact surface was used for anodal stimulation and a SPONSTIM-25 (Neuroelectrics NE, Barcelona, Spain) electrode with 25 cm^2^ contact surface was used as the return electrode (cathode) for bipolar tDCS (Jindal et al., 2015a). For practical considerations based on electrode size and electrode placement by aid of an EEG cap, the electrode positions were defined by the International 10–20 system with fiducials at Nz, Iz, right and left preauricular points for registration with the head model (Jurcak et al., 2007). For subject-specific computation of the electric field and current density induced by tDCS, the 3D electrode coordinates available in the EEG cap along with the fiducials can be individually digitized (e.g., using Fastrack digitizer from Polhemus, USA) and imported in the modeling software for registration with the head model coordinate system. For example, the Montreal Neurological Institute (MNI) coordinate system used by the Colin27 head model (Holmes et al., 1998) uses the anterior commissure as the origin, the X-axis extends from the left side of the brain to the right side, the Y-axis points from posterior to anterior, and Z-axis points from inferior to superior. In this work, we used the MNI coordinates of 10–20 scalp positions given by Okamoto et al. (2004) to define tDCS electrode positions in the SimNIBS software pipeline for the finite element analysis (FEA) using GetDP—a free finite element solver (GetDP: a General Environment for the Treatment of Discrete Problems)^3^. The FEA model used electrostatic volume conductor physics with default (in SimNIBS) material conductivities (in S/m): white matter = 0.126; gray matter = 0.275; CSF = 1.654; bone = 0.01; scalp = 0.465; spongy bone = 0.025; compact bone = 0.008; eye balls = 0.5; eye region = 0.25. The electric field/current density magnitude was used based on prior works (Datta et al., 2011) to determine tDCS-affected brain areas and optimize individual dosing in stroke with MRI-derived realistic head models.

### A computational approach to capture tDCS-affected brain area responses with EEG

A relation between computational model-estimated regional current flow in the brain and experimentally induced changes of functional activation (assessed by functional MRI) has already been shown in a stroke patient (Halko et al., 2011). Electric current density is related to electric field by Ohm's law for static fields. Neuronal excitability changes monotonically with the strength of (weak) electric field magnitude (Datta et al., 2008). In our prior work (Dutta and Dutta, 2013), we have conceptually explored the possibility of using a lead-field based formulation of the electromagnetic reciprocity theorem to reciprocally energize the EEG electrodes in order to target neural sources. In principal accordance, we propose to match the tDCS-affected brain current density distribution with the sensitivity distribution of the EEG Laplacian montage (Gordon and Rzempoluck, 2004), specifically matching their half-sensitivity volumes (Malmivuo et al., 1997), constrained by the International 10–10 system. Therefore, the post-processing step in the ^*^.pro file, generated by the SimNIBS GUI for the FEA with GetDP, was modified to output the tDCS current density distribution at the cortical surface or gray matter (GM) surface. If the electrode montages for tDCS and EEG are identical, e.g., when being reciprocally energized, then the tDCS current flow field can serve as the EEG lead vector field assuming the conductivity tensor as a constant scaling factor (Dutta and Dutta, 2013). In this study, we used a different set of electrodes for EEG so we assumed current sources from the tDCS current density distribution at the nodes of the GM surface and then solved the forward model using the same Colin27 head model to determine the potential distribution at the International 10–10 scalp locations. Then, the best five EEG electrodes in a Laplacian montage from the International 10–10 system were selected using the headModel module of the open-source MoBILAB toolbox (Ojeda et al., 2014) such that their measurement sensitivity distribution best captured tDCS-affected brain areas. The headModel module of the MoBILAB toolbox allows faster Boundary Element Method based computation of the lead field matrix for EEG forward and inverse solutions using OpenMEEG toolbox (Gramfort et al., 2011). The space of orientation free sources was defined on the cortical surface and the lead field for the three volume—scalp, skull, brain—head model was generated for Colin27 (Holmes et al., 1998) using “computeLeadFieldBEM.m” function. The headModel module of the MoBILAB toolbox also uses the Automated Anatomical Labeling (AAL) atlas (Tzourio-Mazoyer et al., 2002) for labeling the cortical surface. When being reciprocally energized (Dutta and Dutta, 2013), sources can be defined on the cortical surface of the target vascular territories based on AAL atlas and the headModel module can be used to quickly optimize multichannel electrode montage for tDCS (Otal et al., 2016) as well as EEG (Väisänen, 2008). When subject's MRI is not available but the EEG electrode positions digitized on the subject's scalp are available then the headModel module of the MoBILAB toolbox provides tools to estimate the head model of the subject by co-registering template's (Colin27) scalp with the subject's sensor positions. Also, b-spline non-linear warping is available to warp subject's scalp topographies to the template's (Colin27) head to compute the inverse solution of the EEG. However, this is not recommended in stroke survivors since Datta et al. (2011) demonstrated significant effects of the lesioned tissue on the current pattern through the brain, including perilesional regions.

### A computational approach to cover tDCS-affected brain area responses with NIRS sensors

To design NIRS probes to cover tDCS-affected brain areas using sources (690 nm: μ*a* = 0.1409∕*mm*, μ*s*′ = 0.9832∕*mm*; 830*nm*:μ*a* = 0.1282∕*mm*, μ*s*′ = 0.7562∕*mm*) and detectors based on our prior work (Jindal and Dutta, 2015), we used the open-source software package called AtlasViewer (Aasted et al., 2015) that provides tools for spatial registration, probe sensitivity computation, and reconstruction of images. The NIRS forward model (and probe sensitivity) is computed by the Monte-Carlo photon transport software, “tMCimg,” available in the AtlasViewer package that computes the probabilistic path of photons from the optode source located at the scalp through the head model tissues to the re-emission at the scalp located optode detectors. We used the Colin27 head model (Holmes et al., 1998) which is provided in the AtlasViewer package along with the International 10–20 system as the reference points for the NIRS probe design. AtlasViewer also provides “iso2mesh”—an image-based 3D surface and volumetric mesh generator comparable to “mri2mesh” tool in the SimNIBS software pipeline (Windhoff et al., 2013)—to generate individual MRI-based head models. The AtlasViewer package allows a probe to be designed, amended, and assessed prior to probe fabrication (Aasted et al., 2015) that we leveraged to design a collinear probe with one source and two detectors. Increasing the source-detector (SD) separation past 2 cm monotonically increases sensitivity to brain tissue; diminishing returns appear to begin at around 4–5 cm (Strangman et al., 2013). In order to identify systemic interference using short-separation NIRS measurements (Sood et al., 2015a), a short SD separation was incorporated in the probe design to explicitly sample extra-cerebral tissues. The optimum short SD separation is 8.4 mm with the Colin27 head model (Brigadoi and Cooper, 2015). Therefore, a probe design of short SD separation of 8 mm and long SD separation of 2–4 cm was decided to best cover tDCS-affected brain areas. For optimal NIRS probe placement with AtlasViewer package, the anchor points were defined at the F3, Fz, C3, Cz locations of the International 10–20 system and the length of the springs were chosen to ensure that the collinear NIRS probe warps along the arc joining F3 and Cz on the scalp surface. The probe sensitivity was defined using the Monte-Carlo (MC) photon transport software “tMCimg” (Boas et al., 2002) available in the AtlasViewer package. Initial rapid assessment of the probe placement and sensitivity was performed with 1e6 photons. More accurate results were obtained with 1e8 photons to evaluate the final probe design. This time-consuming MC simulation generates the forward matrix that represents the spatial sensitivity profile of each measurement channel to cortical absorption changes. A graphical processor unit can substantially speed up the simulation by more than 100 × using mesh-based MC simulation (Fang, 2010). The NIRS forward model is identified for the head volume where AtlasViewer projects the volumetric sensitivity in the gray matter onto the surface of the pial matter and also implements the AAL atlas (Tzourio-Mazoyer et al., 2002) for localizing the brain region of interest. In fact, NIRS signals in adult humans are strongly biased toward the outermost 1–1.5 cm of the intracranial space (Strangman et al., 2013). Registration of this head model to a subject can be achieved using affine transformation in the AtlasViewer with fiducials 3D digitized at Nz, Iz, Cz, right and left preauricular points. It is also essential to incorporate optical properties representing heterogeneously lesioned individual brains to build realistic individual head models, especially, for the reconstruction of images of the measured brain activation patterns in stroke survivors.

### Computational modeling of interactions between the neuronal and hemodynamic responses to tDCS in the lesional and contralesioned hemispheres

The computational modeling approach aims to examine the combined neurovascular origin of the brain activation signals. Therefore, we aim to combine the EEG and NIRS signals in a single system model to understand the relationship between the signals during periods of tDCS-evoked activation (Dutta et al., 2015). In our clinical study (Jindal et al., 2015b) that was limited by the specifications of a custom-built low-cost NIRS data acquisition system (Jindal and Dutta, 2015), we placed the probe on the forehead to prevent that hair follicles affect the readings. This procedure was supported by computational modeling that showed an electric field of ~0.15 V/m (roughly half of the maximum ~0.33 V/m) in the gray matter of the forehead (Fp1-F7 region) due to F3 anodal and Cz cathodal tDCS (Jindal et al., 2015a). For this study, we followed a randomized block design of experiments. tDCS was turned ON for 50 s with 5 s ramp-up and 5 s ramp-down, which was repeated 15 times in random order for each hemisphere (ischemic stroke was restricted to a single hemisphere) with 60 s OFF periods in-between. Eyes-open block-averaged resting-state NIRS oximeter measurements were conducted just above each eyebrow and below the F3 and F4 sites using adult SomaSensor (SAFB-SM, INVOS, USA) at a sampling frequency of 10 Hz. The eyes-open resting-state NIRS signal corresponding to regional cerebral hemoglobin oxygen saturation (rSO2) in the middle 50 s OFF period (rejecting 5 s leading and 5 s trailing ends of 60 s) was analyzed, i.e., 50 s OFF period for the left and 50 s OFF period for the right hemispheres repeated 15 times in random order. The eyes-open resting-state EEG (StarStim, Neuroelectrics, Spain) was recorded at 500 Hz from the nearby electrodes F1, FC3, F5, F2, FC4, F6 (international 10–10 system), synchronized with rSO2 time-series by sending markers from a custom-made oximeter (Jindal and Dutta, 2015) using TCP/IP. The EEG in the OFF periods was pre-processed using EEGLAB functions. Artifactual (“non-stereotyped” or “paroxysmal” noise) epochs were removed following visual inspection (Delorme and Makeig, 2004), and then the average EEG from F1, FC3, F5 sites was subtracted from the F3 site of the left hemisphere and average the EEG from F2, FC4, F6 sites was subtracted from the F4 site for the right hemisphere EEGs.

We assumed focal unihemispheric effects of anodal tDCS for 50 s with 5 s ramp-up and 5 s ramp-down that was repeated 15 times in random order for each hemisphere. Empirical Mode Decomposition (EMD) into a set of intrinsic mode functions (IMFs) was performed using the Hilbert-Huang Transform (HHT) for rSO2 (called IMF_NIRS_) and EEG (called IMF_EEG_) time-series for each hemisphere. Generally, the first IMF contains the highest frequency components and the oscillatory frequencies decrease with increasing IMF index. IMFs containing peak power >50 Hz were discarded and then the log (base-10) transformed mean power of IMF_EEG_ data was computed (see Dutta et al., 2015 for details). The IMF_EEG_ mean-power time-series was down-sampled to 10 Hz to match the sampling frequency of the IMF_NIRS_ time-series. Cross correlation calculations (Pfurtscheller et al., 2012) were performed between each OFF time-series for IMF_EEG_ mean-power and IMF_NIRS_. Here, the number of lag of ±20 s (Matlab function “crosscorr”) was selected based on prior work (Pfurtscheller et al., 2012) which identified short-lasting coupling with lead/lag < 20 s. Cross-correlation values within ±3 standard deviations for the estimation error were set to zero assuming the signals to be uncorrelated.

## Illustrative results

### A computational approach to target vascular territories with tDCS

**Figure 1:**
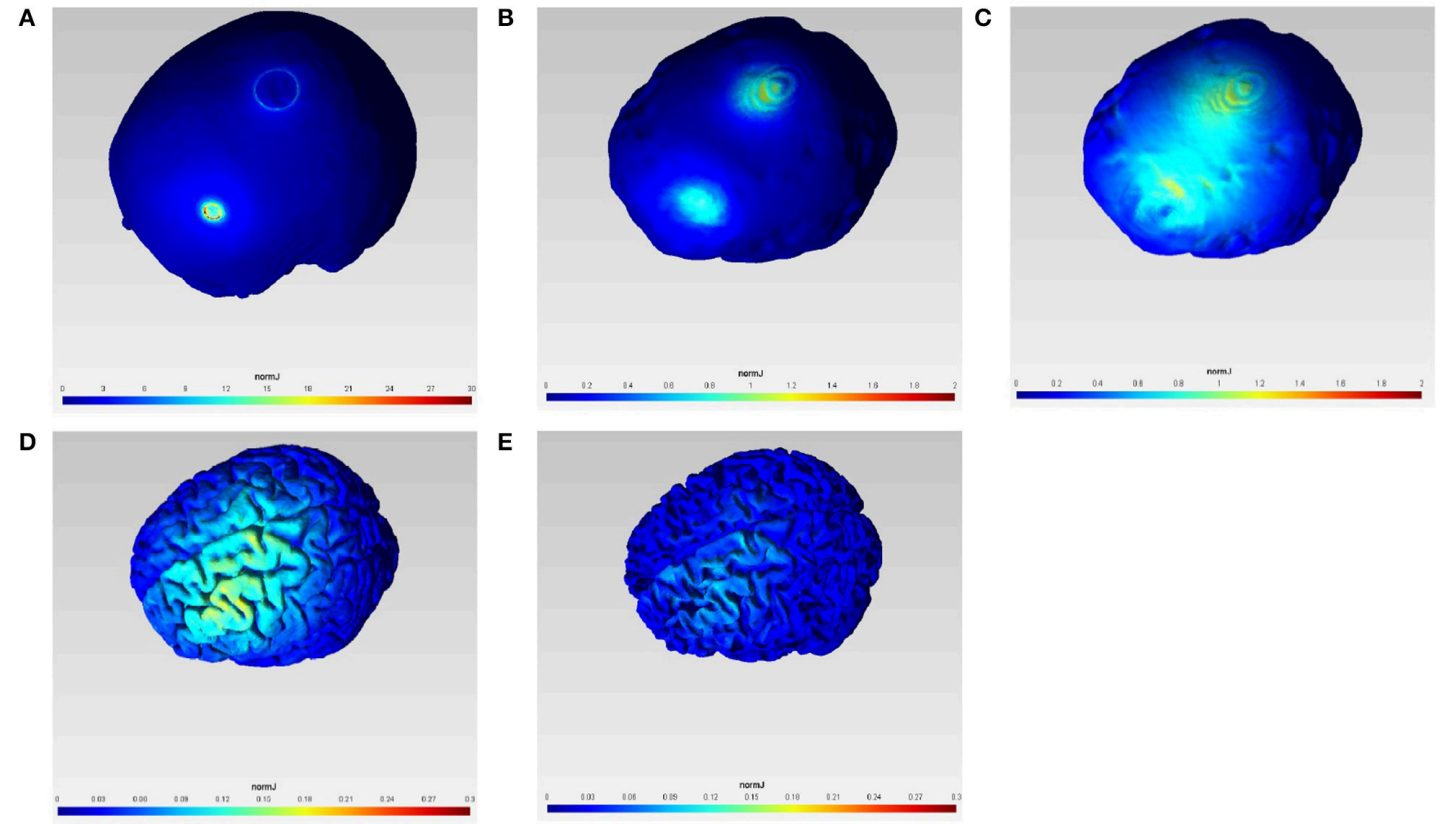
**Current density magnitude (normJ) at the scalp surface (A), skull surface (B), CSF surface (C), gray matter surface (D), and white matter surface (E) with 1 mA F3 anodal and Cz cathodal tDCS**.

Figure 1 shows the current density magnitude at the scalp surface, skull surface, CSF surface, gray matter surface, and white matter surface with 1 mA F3 anodal and Cz cathodal tDCS which was selected in order to target primarily the outer convexity of the brain MCA territory. The spread of current between the anode and the cathode is visible in the highly conductive CSF volume (see Figure 1C) which covers the outer convexity of the brain MCA territory in the gray matter surface plot (Figure 1D). However, the scale of the current density magnitude reduces by one-hundredth from the scalp surface plot (Figure 1A) to gray matter (Figure 1D) and white matter (Figure 1E) surface plots. We computationally confirmed primarily unihemispheric effects of anodal tDCS in the gray and white matter with our electrode montage.

### A computational approach to capture tDCS-affected brain area responses with EEG

**Figure 2:**
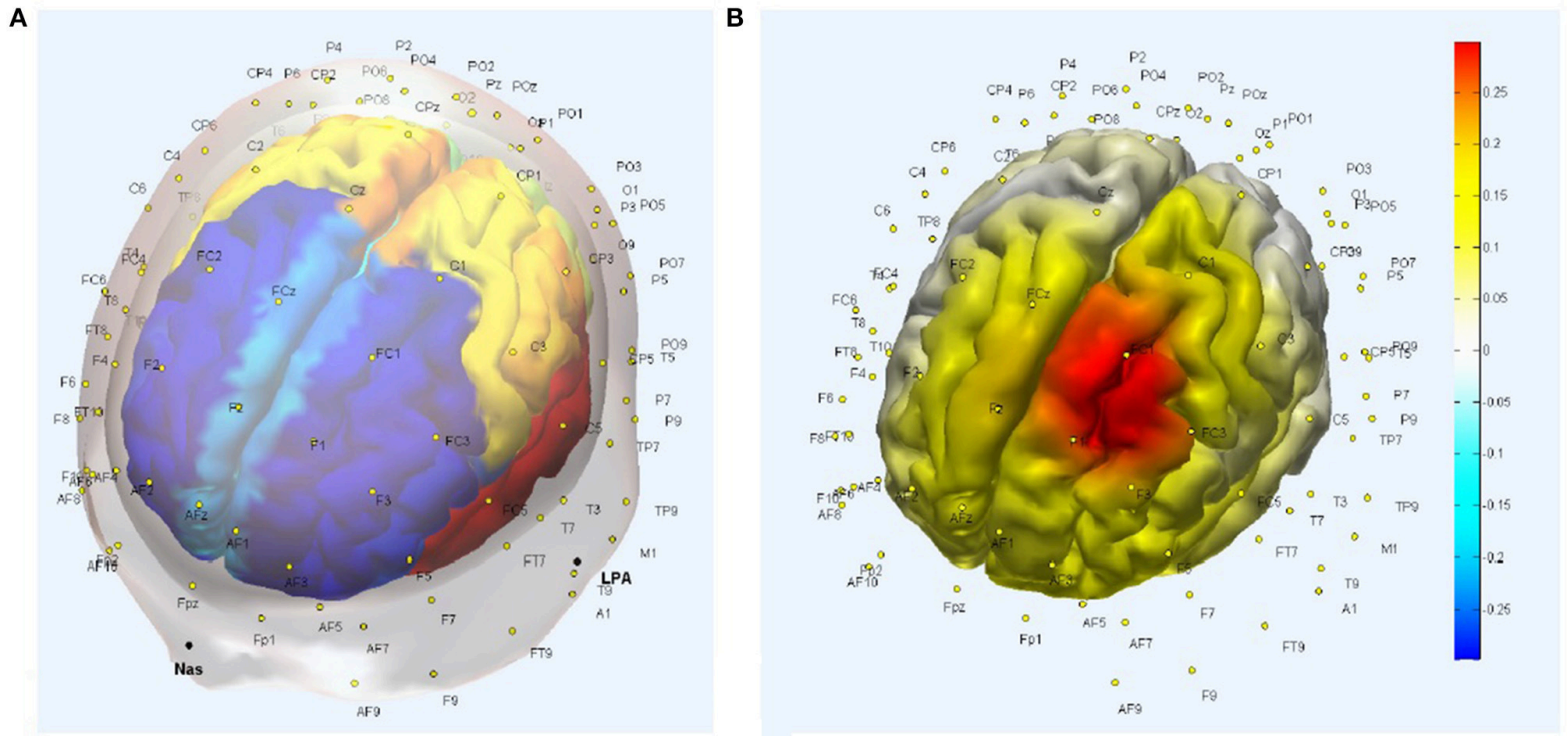
**(A)** Head model with Automated Anatomical Labeling (AAL) atlas and EEG scalp locations. **(B)** Measurement sensitivity distribution of the Laplacian spatial filter—FCz, C1, FC3, F1, and FC1—at the gray matter surface that covers primarily middle frontal and superior frontal gyrus.

The five best EEG electrodes in the Laplacian montage from the International 10–10 system selected to capture the tDCS affected gray matter response (Figure 1D) were FCz, C1, FC3, F1, and FC1, as shown in Figure 2A of the head model with AAL atlas color labeling and EEG scalp locations. Here, FCz, C1, FC3, F1, and FC1 create a Laplacian spatial filter whose measurement sensitivity distribution at the gray matter surface is shown in Figure 2B. The measurement sensitivity distribution of the Laplacian spatial filter was implemented to capture the tDCS-affected gray matter (see Figure 1D) response primarily in the middle frontal and superior frontal gyrus of Brodmann area 6 (Koessler et al., 2009).

### Computational approach to cover tDCS-affected brain area responses with NIRS sensors

**Figure 3:**
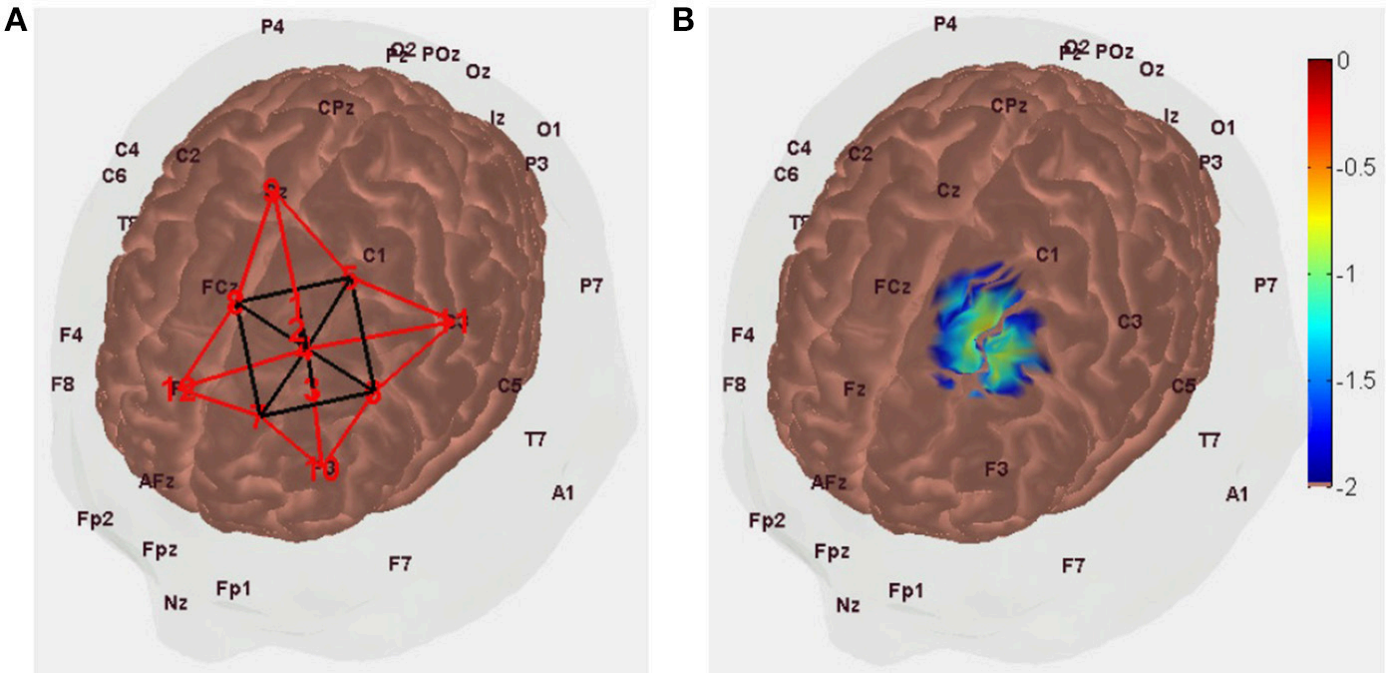
**(A)** NIRS probe placement along the arc joining Cz and F3 scalp locations with the optode source (labeled “1”) 1.5 cm toward Cz from FC1 (labeled “4”) and short separation (labeled “2”) and long separation (labeled “3”) detectors are 8 mm and 3 cm toward F3 from optode source (i.e., labeled “1”). **(B)** Measurement sensitivity distribution of NIRS probe at gray matter surface that covers primarily middle frontal and superior frontal gyrus.

A NIRS probe design with short SD separation of 8 mm and long SD separation of 3 cm was found optimal with regard to covering tDCS-affected gray matter (see Figure 1D), as shown in Figure 3A.

**Figure 4:**
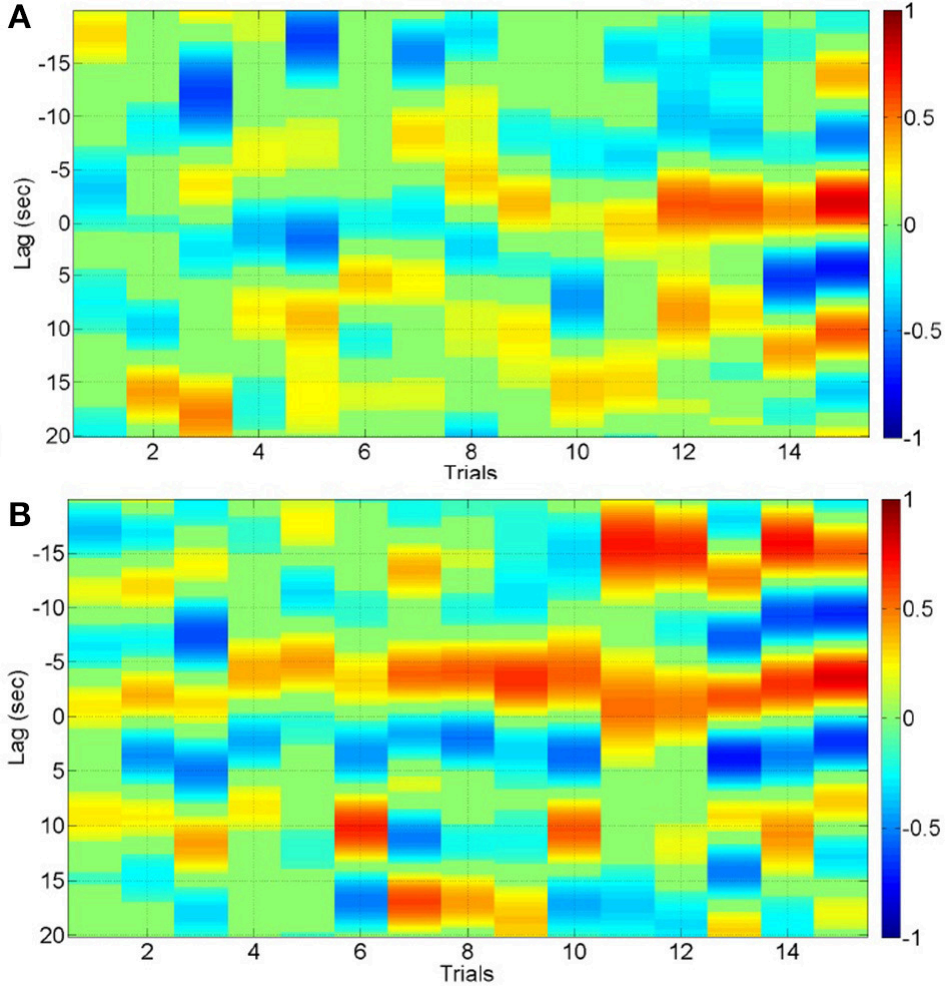
**Cross correlation function for the 3rd IMF with lead/lag < 20 s for 15 trials of lesional hemisphere (A) and contralesional hemisphere (B)**.

In Figure 3A, the NIRS probe is placed along the arc joining Cz and F3 scalp locations, with the probe midpoint (labeled “4”) at FC1 so the optode source (labeled “1,” MNI coordinate: [−16, 16, 71]) is 1.5 cm toward Cz from FC1 and short separation (labeled “2,” MNI coordinate: [−17, 20, 62]) and long separation (labeled “3,” MNI coordinate: [−18, 24, 45]) detectors are 8 mm and 3 cm toward F3 from the optode source (i.e., labeled “1”). The measurement sensitivity distribution of the NIRS probe is shown in Figure 4B to capture the tDCS-affected gray matter (see Figure 1D) response primarily in the middle frontal and superior frontal gyrus of Brodmann area 6 (Koessler et al., 2009).

### Computational modeling of interactions between the neuronal and hemodynamic responses to tDCS in the lesional and contralesional hemispheres

The cross correlation function with lead/lag < 20 s for the 3rd IMF—the most common across significant positive and negative cross-correlations—is shown in Figure 4A for the 15 trials of the lesional hemisphere (LH) and in Figure 4B for the 15 trials of contralesioned hemisphere (CH). The stimulation-and-recording order was—CH, CH, CH, LH, LH, CH, LH, LH, LH, CH, CH, CH, CH, LH, CH, LH, LH, LH, LH, CH, LH, CH, CH, CH, LH, CH, CH, LH, LH, LH. The cross-correlation values are shown according to the color bars. Both positive and negative cross-correlations can be found at different lead/lag. For the CH hemisphere, there is a strong positive correlation with a lag of about −15 s (i.e., IMF_NIRS_ leads IMF_EEG_ mean-power) starting at the 11th trial, i.e., after cumulative 550 s of anodal tDCS.

## Discussion

In this methods paper, we investigated the bipolar tDCS montage from our prior works with a software pipeline incorporating freely available software packages to design a NIRS-EEG probe for joint imaging of tDCS-evoked response. The software pipeline was applied in a ischemic MCA stroke case based on our prior works (Dutta et al., 2015; Jindal et al., 2015b). The direction of the tDCS effects on cortical excitability are polarity specific. Anodal tDCS increases and cathodal stimulation decreases cortical excitability, within specific limits of stimulation duration and intensity (Stagg and Nitsche, 2011; Batsikadze et al., 2013; Monte-Silva et al., 2013). Therefore, we postulate an alternate mechanism that tDCS evoked response may not only be due to tDCS effects on neuronal tissue, but may also be caused by its direct effects on glial cells (Monai et al., 2016) and the vascular tissue (Dutta, 2015; Pulgar, 2015). Pial arteries and arterioles contain perivascular nerves within their adventitial layer that can be affected by tDCS. In support, Figure 1C shows that the magnitude of tDCS current density (normJ) in the CSF (normJ) that surrounds pial vessels (Cipolla, 2009) is almost 10 times of that in the white matter. Furthermore, it is postulated that the electric field direction (e.g., perpendicular to the gray matter surface) is more conducive in polarizing neurons in the close vicinity of the stimulating electrode in the gray matter while the large astrocytic network may be more susceptible to the widespread electric field strength where the peak usually lies in between the stimulation and return electrodes. Indeed, polarity specific effects of tDCS on cortical excitability alterations have been shown (Stagg and Nitsche, 2011) which lends to our hypothesis that the orientation of the electric field may be more relevant for neuronal stimulation, and therefore the optimal location for neuronal stimulation may be different than that for astrocytic stimulation. Here, widespread spatiotemporal bidirectional interactions between the neuronal and hemodynamic cerebral responses are postulated (Dutta, 2015), with an important role of astrocytes within the NVU where the transition between vasoconstriction and vasodilatation was observed in a single vessels by varying the stimulation intensity (Tsytsarev et al., 2011). Stimulation of astrocytes raises calcium in the end-feet that can have a vasoactive effect on parenchymal arterioles. Dilation or constriction depends on the level of calcium (Mulligan and MacVicar, 2004). Indeed, differences of calcium dynamics are proposed to result in different effects of specific tDCS protocols (Stagg and Nitsche, 2011). For example (Fricke et al., 2011), 5-min anodal tDCS increases motor cortex excitability for about 5 min, while cathodal tDCS of the same duration reduces excitability. Increasing the duration of tDCS to 10 min prolongs the duration of the effects, and if the two 5-min periods of tDCS are applied with a 30-min break between them, then the effect of the second period of tDCS was identical to that of the 5-min stimulation alone. However, if the break was only 3 min, then the second session had the opposite effect compared to 5-min tDCS given alone. Here, we postulate that NIRS-EEG online portable neuroimaging measurements may be used to estimate cortical excitability (Jindal et al., 2015a) by simulating the NVC (Jindal et al., 2015b) that may lend to closed-loop dosing of tDCS (Dutta, 2015).

NIRS-EEG joint imaging to analyze NVC requires the development of a multi-modal sensor montage as well as computational tools that can parameterize NVU dynamics based on the measured data (Huneau et al., 2015). Figure 1 shows the brain areas affected by the implemented bipolar tDCS montage. The NIRS-EEG sensor montage needs to be designed to be sensitive to the tDCS affected brain areas. The EEG electrode montage can act as a spatial filter. The EEG forward model allows computation of the lead-field as a function of dipole position, thereby showing the spatial variation of the sensitivity of the montage (Gordon and Rzempoluck, 2004), as shown in Figure 2B. In fact, the second spatial derivative of the recorded surface potentials during a evoked response represent the magnitude of the transcranial current flow leaving (sinks) and entering (sources) the scalp (Perrin et al., 1989) where the maxima and minima of the sink-source scalp pattern can be used to place anodes (at sources) and cathodes (as sinks) to target the neural source of the evoked response (Dutta and Dutta, 2013). In our prior work, we have conceptually explored the possibility of using a lead-field based formulation of the electromagnetic reciprocity theorem to reciprocally energize EEG electrodes in order to target neural sources with tDCS (Dutta and Dutta, 2013). Here, the lead-field in EEG source analysis is related to the tDCS current flow field via its product with the conductivity tensor for an identical electrode montage. An EEG measurement sensitivity analysis (shown in Figure 2B) is related to tDCS electric field/current density modeling (shown in Figure 1). Therefore, the Laplacian spatial filter—FCz, C1, FC3, F1, and FC1, shown in Figure 2, can be reciprocally energized using 4 × 1 HD-tDCS (Villamar et al., 2013) for more focal (unihemispheric) stimulation of the gray matter surface covering primarily middle frontal and superior frontal gyrus (see Figure 2B). Also, NIRS imaging in conjunction with EEG to analyze neurovascular coupling requires development of NIRS-EEG joint-imaging sensor montage that is sensitive to the same targeted region of the brain (Giacometti and Diamond, 2014), which was identified using the NIRS forward model (Aasted et al., 2015). The preferred NIRS probe placement and its measurement sensitivity distribution at the gray matter surface is shown in Figure 3. Moreover, the ill-posed inverse of the forward model may be used to compute scalp level topographic sensor maps where beam forming approaches may be used to optimize multichannel EEG methods (Väisänen, 2008) in conjunction with multichannel tDCS (Otal et al., 2016) using the electromagnetic reciprocity theorem (Dutta and Dutta, 2013).

In our prior work (Dutta et al., 2015; Jindal et al., 2015b), we developed a low-cost NIRS data acquisition system (Jindal and Dutta, 2015) and conducted a clinical study with patients with MCA stroke. However, the *ad hoc* probe placement was limited to the forehead so that the hair follicles do not affect the readings. Also, NIRS imaging during tDCS requires identification of systemic interference using short-separation NIRS measurements (Sood et al., 2015a) to explicitly sample the extra-cerebral tissues response. We found interhemispheric laterality in the systemic interference as well as mean cerebral hemoglobin oxygen saturation evoked by anodal tDCS in some stroke subjects (Sood et al., 2015a). In those subjects, primarily with Large Artery Atherothrombosis (LAA), we hypothesized that—since LAA leads commonly to stenosis at the bifurcation of the carotid artery (carotid stenosis) the internal carotid artery (ICA) that supplies blood to the brain and the external carotid artery (ECA) that supplies blood to the head and neck (such as face, scalp, etc.)—interhemispheric laterality of a carotid stenosis may lead to laterality in the hemodynamic response to tDCS both at the brain tissue (due to ICA) as well as at the extra-cerebral tissues (due to ECA). This results in short SD separation measurements primarily related to ECA while the longer SD separation measurements primarily related to ICA which is of primary interest in human stroke studies (Sood et al., 2015a). In this study, we further noticed that as we increased the tDCS current density to around 0.6 A/m^2^, the measurement by the short separation SD pair saturated around 0.3 A/m^2^, while the measurement by the long separation SD pair continued to increase, albeit slightly, from 0.3 to 0.6 A/m^2^. We hypothesize that the signals evoked at the lower tDCS current density are primarily derived in the extra-cerebral tissues for both long and short separation SD pairs. These can be used for calibration using general linear model (GLM) approach to adjust for short separation SD measurement in order to improve the probe sensitivity to brain tissue (Yücel et al., 2015). This tDCS based calibration technique should work for any probe placement when compared to our earlier approach where we used an anterior temporal artery tap to identify systemic interference using short-separation NIRS measurements that worked well for a forehead probe (Sood et al., 2015a).

It should be noted that tDCS can have direct effects on glial cells (Monai et al., 2016) and smooth muscles of blood vessels (Pulgar, 2015), and cerebral autoregulation mechanisms can ensure that the blood flow is maintained during changes of perfusion pressure. This can lead to multi-timescale cross-talk and resulting complex non-linear dynamics (Jolivet et al., 2015). Figure 4 shows the results from cross correlation calculations where both positive and negative cross-correlations were found at different lead/lag. In the contralesional hemisphere, there was a strong positive correlation with a lag of about -15 s after a cumulative 550 s of anodal tDCS. In case of positive cross-correlations, primarily a negative lag was found, i.e., IMF_NIRS_ leads IMF_EEG_ mean-power. In our current study, we used a block design where block average over all stimulus onsets is possible for the cross correlation function. However, a more robust deconvolution approach (Peng et al., 2016) may be appropriate to continuously monitor the hemodynamic signal evoked by tDCS perturbation where robust system identification techniques, e.g., continuous-time autoregressive (ARX) model (Sood et al., 2015b), may be applied to capture the coupling relation between IMFs of regional cerebral hemoglobin oxygen saturation and the log-transformed mean-power time-series of IMFs for EEG from the lesional and contralesional hemispheres. Here, subject-specific alterations of ARX poles and zeros with different dead time may be relevant for diagnosing dysfunction in cerebrovascular occlusive disease (Phillip et al., 2013).

## Author contributions

AD has substantially contributed to the conception and design of methods for this work. AD and DG have drafted the work and have reviewed it critically. All authors have approved the final version prior to submission and are in agreement.

## Funding

This research work was partly supported by the Institut National de Recherche en Informatique et en Automatique (INRIA), France. The publication of this article was funded by the open access fund of the Leibniz Association.

### Conflict of interest statement

The authors declare that the research was conducted in the absence of any commercial or financial relationships that could be construed as a potential conflict of interest.

## Footnotes

^1^http://www.who.int/cardiovascular_diseases/resources/atlas/en/ [Accessed November 8, 2014].

^2^http://simnibs.de/version2/mri_sequences [Accessed March 26, 2016].

^3^http://getdp.info/ [Accessed March 26, 2016].
